# Global, regional, and national burdens of facial fractures: a systematic analysis of the global burden of Disease 2019

**DOI:** 10.1186/s12903-024-04048-5

**Published:** 2024-02-28

**Authors:** Ze-Xing Zhang, Long Xie, Zhi Li

**Affiliations:** 1 State Key Laboratory of Oral & Maxillofacial Reconstruction and Regeneration, Key Laboratory of Oral Biomedicine Ministry of Education, Hubei Key Laboratory of Stomatology, School & Hospital of Stomatology, Wuhan University, Wuhan, 430072 China; 2 Department of Oral and Maxillofacial Surgery, School and Hospital of Stomatology, Wuhan University, 237 Luoyu Road, Wuhan, 430079 China

**Keywords:** Disease burden, Incidence, Prevalence, Years lived with disability, Facial fractures

## Abstract

**Background:**

The incidence of facial fractures has undergone tremendous changes in recent years as a result of socio-economic development and aging populations. Currently, there is a lack of updated and comprehensive analyses of global trends and causes of facial fractures. The Global Burden of Disease (GBD) database is a product of a global research organization used to quantify the global impact of hundreds of diseases, injuries, and risk factors. The aim of this study was to update global burden of facial fractures from 1990 to 2019 by using the GBD2019.

**Materials and methods:**

The present study extracted the global incidence, prevalence, and years lived with disability (YLDs) for facial fractures, as well as the age-standardized rates (ASRs) of these variables using the Global Burden of Disease (GBD) 2019 database. The estimated annual percentage change (EAPC) was used to assess the trends of ASRs.

**Results:**

Between 1990 and 2019, the incidence of facial fractures increased from 8,943,707 to 10,676,340, but the age-standardized incidence rate (ASIR) decreased from 161.5 to 138.8 per 100,000. Prevalence and YLDs exhibited the same trend as incidence. Over the 30 years, the incidence of facial fractures was consistently greater in males than in females. However, females aged ˃ 75 years had higher fracture incidence rates than males aged ˃ 75 years in 2019. The leading cause of facial fractures was falls, and both the age-standardized prevalence rate (ASPR) and age-standardized years lived with disability rate (ASYR) of falls increased with age.

**Conclusion:**

Facial fractures still represent a significant burden to the world. Incidence, prevalence and YLDs all showed increasing trends, while ASRs decreased gradually from 1990 to 2019. Enhancing the quality of facial fractures data is helpful for monitoring the burden of facial fractures.

**Supplementary Information:**

The online version contains supplementary material available at 10.1186/s12903-024-04048-5.

## Background

Injuries impose a colossal burden globally, causing multiple disabilities and deaths [1]. Fractures are a common type of nature of injuries, with 76.4 million occurring in 2019 alone [2]. The incidence of facial fractures was approximately 10.7 million cases in 2019, which was almost one seventh of bone fractures globally. Unfortunately, facial fractures remain largely neglected [3]. In some facial fracture cases, they could be able to be managed on an ambulatory basis. However, when facial fractures are accompanied by other related injuries or complications, such as brain injury or multiple fractures, it usually leads to expensive treatment costs and long-term hospitalizations [4]. Besides, facial fractures can directly affect oral health [5], especially in cases of penetrating injuries, with sequelae ranging from single tooth damage to complete tooth loss. These fractures often require restoration of damaged and missing teeth, leading to extremely high treatment costs [6, 7].

Previous studies of facial fractures are limited to reports of simple descriptive statistics [8, 9]. The incidence of facial fractures has undergone tremendous changes in recent years as a result of socio-economic development and aging populations [10]. Economic development means more diverse and sound detection methods, such as CT (computed tomography) and three dimensional (3D) reconstruction, which can diagnose facial fractures more comprehensively and conveniently, avoiding misdiagnosis and missed diagnosis caused by previous use of X-rays [11]. At the same time, the improvement of people’s living standards has led them to pay more attention to their physical health and facial aesthetics. These factors all increase the detection rate of facial fractures. Compared with young men, falls are the main cause of facial fractures in the elderly. However, the most common cause of facial fractures in young men is assault [12]. There is no doubt that the aging population has a high incidence rate of facial fractures caused by falls. Currently, there is a lack of updated and comprehensive analyses of global trends and causes of facial fractures.

The Global Burden of Disease (GBD) database is a global database for disease burden assessment [1, 13]. GBD 2019 tracks and systematically evaluates data for 369 diseases and injuries in 204 countries and territories. Additionally, it includes an improved estimation process and more comprehensive data sources compared to GBD 2017. Based on the data of facial fractures from GBD 2019, this study aims to systematically assess the global burden, trend, etiologies, and influence factors of facial fractures to help formulate more reasonable policies and demonstrate the need for such policies.

## Methods

### Overview

GBD 2019, which expanded and updated GBD 2017, was a systematic review based on 204 countries and territories, including 369 diseases and injuries and 87 risk factors from 1990 to 2019. All data for this study were retrieved using the Global Health Data Exchange query tool (http://ghdx.healthdata.org/gbd-results-tool). And all data was contacted by the Institute for Health Metrics and Evaluation (IHME). The data published in GBD2019 is in accordance with the Guidelines for Reporting Accurate and Transparent Health Estimates (GATHER) [1].

### Disease definition

GBD defines fracture as the nature of injury, not the cause of the disease. The GBD definition of facial fractures was based on the International Classification of Disease and Injuries (ICD). Consistent with ICD9 code 802 and ICD10 codes S02.2, S02.3, S02.4, S02.5, S02.6, and S02.7, the specific definition of facial fracture cases in GBD includes nasal, orbital, mandibular, maximal, and other facial bone fractures.

### Data processing and relevant parameter

A bayesian meta-regression tool called Bayesian meta-regression version 2.1 (DisMod-MR 2.1) was used to estimate from complicated data to a series of incidence, prevalence, mortality and every cause, ages level and location and so on.

The causes were classed into four levels, and among them, most of data came from hospital record, injury surveillance, civil registration and vital statistics. The etiology classification of fractures uses the most specific causes (such as road traffic injuries or falls). A straightforward approach for collecting data on bone fractures was published in a previous study [2]. In a word, firstly, they used clinical record to calculate proportions of causes and corresponding nature of injury like fracture. If the fracture happened on many sites of body, it would base on disability weight to choose the most serious site. The weight was come from survey and of household and web, which could show the relative severity of the fracture site. And they used a multinomial regression to make sure the proportion of all consequence of injury sum to 1.

### Data sources

The Global Burden of Disease (GBD) database is a product of a global research organization used to quantify the global impact of hundreds of diseases, injuries, and risk factors. GBD tried to use all available data sources to provide comprehensive and authoritative data, including civil registration and vital statistics, surveys from household, and records of hospital [14]. Detailed methods for the estimation of facial fracture data have been previously reported [2]. All data, including incidence, prevalence, and years lived with disability (YLDs) of different ages and overall for facial fractures between 1990 and 2019 were extracted to assess the global burden of facial fractures. Besides, age-standardized rates (ASRs) of these three variables also downloaded from GBD website. GBD uses standard population structure to estimate number and age-standardized rates date per 100,000. And they drew 1,000 values from the posterior distribution, values from 25th to 975th of final estimated were regarded as 95% uncertainty intervals (UI). The socio-demographic index (SDI) for each country was obtained from the GBD official website (https://vizhub.healthdata.org/gbd-results/). SDI is a comprehensive index that calculates three aspects of data according to per capita income, average education level, and pregnancy rate under 25 years old, and is highly correlated with health outcomes. The SDI scale ranges from 0 to 1 and is divided into five grades: low (0.0–0.2), low-medium (0.2–0.4), medium (0.4–0.6), high-medium (0.6–0.8), and high (0.8–1.0), with 0 indicating the lowest level of health-related development. Finally, the disease burden of facial fractures was further analyzed by age, cause, and sex.

### Data analysis

Estimation methods for the disease burden of facial fractures have been described in previous articles [1]. ASRs (per 100,000 population) of incidence, prevalence, and YLDs were obtained directly from the GBD results tool. Estimated annual percentage changes (EAPCs) were used to describe the changing ASR trends. Assuming a generalized linear relationship between the natural logarithm (ln) of ASR and time, *y = α + βx + ε*, where *y* refers to ln (ASR), *x* refers to the calendar year, and *ε* is the standard deviation. In this formula, β decides the trend of ASR. EAPC was calculated as 100 *×* (exp (*β*) *−* 1), and its 95% certainty intervals (CI) for EAPC was gained by using a linear model. When EAPC and the minimum limit of its 95% CI were > 0, ASRs showed an increasing trend; when EAPC and the minimum limit of its 95% CI were ˂0, ASRs showed a decreasing trend. All statistical analyses were performed using the open-source software R (version 4.1.3; The R foundation, Vienna, Austria). When *p* < 0.05, it can be considered that the two are statistically significant.

## Results

### Incidence

**Fig. 1:**
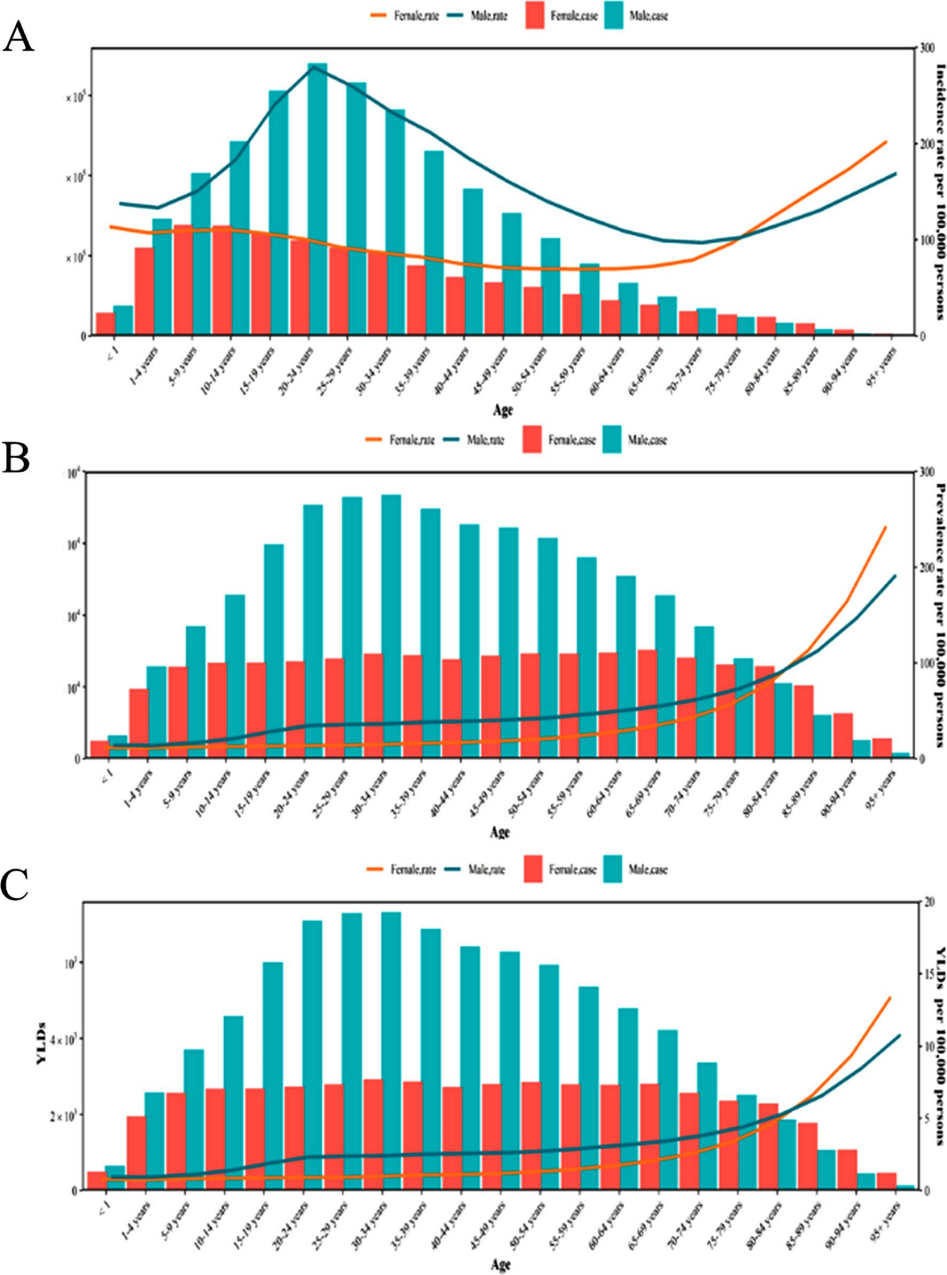
Global disease burden of facial fracture by age and sex in 2019. (**A**) Incidence; (**B**) prevalence; (**C**)YLDs

Between 1990 and 2019, the global incidence of facial fractures increased from 8943.7 thousand (95% UI, 7120.5–11371.2 thousand) to 10676.3 thousand (95% UI, 8504.3–13455.6 thousand), an increase of 19.4%. In contrast, Age-standardized incidence rate (ASIR) gradually decreased over these 30 years from 161.5 (95% UI, 128.7–204.7) per 100,000 in 1990 to 138.8 (95% UI, 110.6–174.8) per 100,000 in 2019, with an EAPC of − 0.5(95% CI: −0.4 to − 0.6) (Appendix Table 1). The male-to-female incidence ratio remained unchanged at 2:1 during these three decades, but showed a slight downward trend from 2010 onwards (Fig. S1A). Incidence among males peaked at the age of 15–34 years. Males had a higher age specialization incidence rate than females until the age of 75 years, and then the age specialization incidence rate of females surpassed that of males after the age of 75 years (Fig. 1A).

**Fig. 2:**
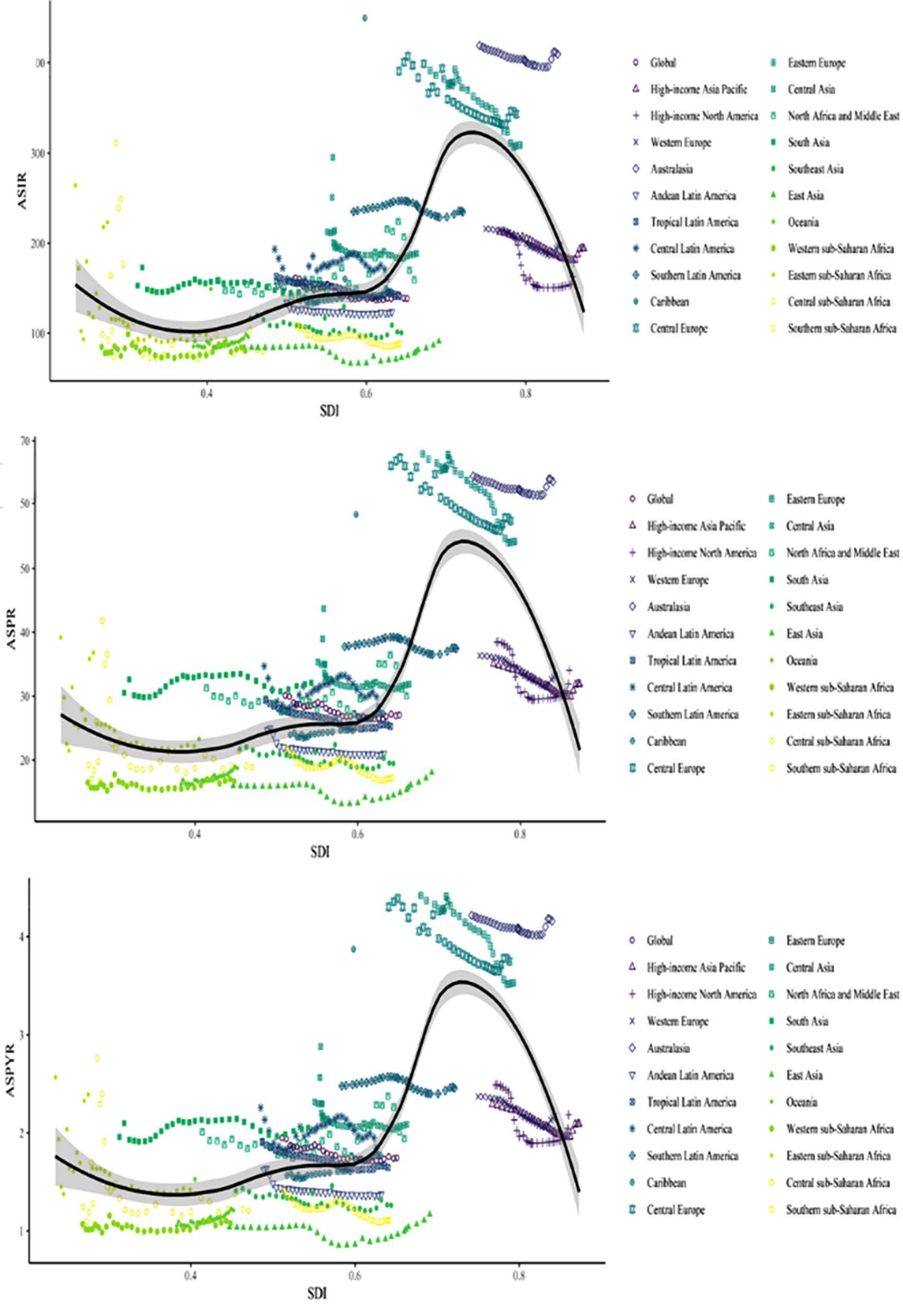
Global disease burden of facial fractures for both sexes in 204 countries and territories. Note: (**A**) ASIR of facial fractures in 2019; (**B**) The ASPR of facial fractures in 2019; (**C**) The ASYR of facial fractures in 2019

**Fig. 3:**
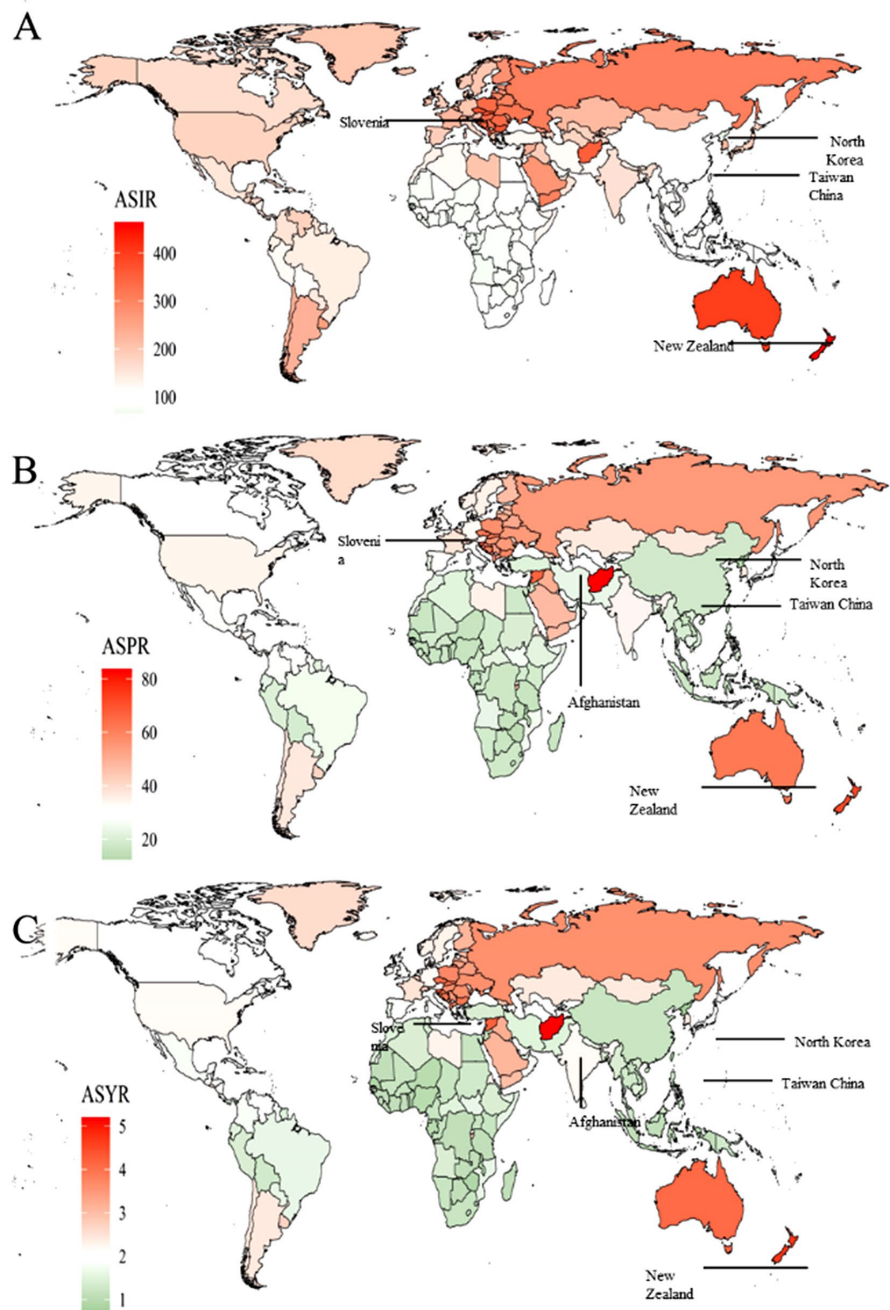
Age-standardized rates of facial fractures among regions based on SDI in 2019. *Note* (**A**) ASIR; (**B**) ASPR; (**C**)ASYR

In 2019, New Zealand, Slovenia had the highest ASIRs, while North Korea and the Taiwan region had the lowest ASIRs (Fig. 2A, Appendix Table 2). There was a significant positive correlation between SDI and ASIR. The ASIR showed a gradual upward trend in the regions with medium SDI, a significant increase in the regions with high-medium SDI, and a significant decrease in regions with high SDI. The ASIRs in Central and Eastern Europe were well above the average. High SDI regions had higher ASIRs, but with a downward trend (Fig. 3A). New Zealand, Slovenia had significantly higher ASIRs than the overall trend, whereas the ASIRs for North Korea and Taiwan were considerably lower (Fig. S2A).

From 1990 to 2019, although the incidence showed an increasing global trend, it decreased in five regions. The developed regions of Central and Eastern Europe showed the greatest decreases and had relatively high ASIRs. The greatest increase in ASIR was seen in developing regions, including Oceania, North Africa, the Middle East, and the Caribbean (Appendix Table 1). Qatar had the most significant increase in the incidence (Fig. S3A). Furthermore, Syria had the highest EAPC, which far exceeded the world average. Conversely, Liberia had the lowest EAPC (Fig. S4A, Appendix Table 2).

### Prevalence

Globally, the prevalence of facial fractures increased by 41.84% from 1502.8 thousand (95% UI, 1242.6–1823.6 thousand) in 1990 to 2131.6 thousand (95% UI, 1815.7–2509.6 thousand) in 2019. However, age-standardized prevalence rate (ASPR) showed a slight downward trend, from 30.1 (95% UI, 25.4–35.9) per 100,000 to 27.0 (95% UI, 23.0–31.9) per 100,000, while there was a negative EAPC of − 0.4 (95% CI: −0.3 to − 0.5) (Appendix Table 3). The prevalence of facial fractures in males has been approximately twice that in females over the past 30 years (Fig. S1B). The age specialization prevalence rate in males was higher than in females up to the age of 80 years, after which the age specialization prevalence rate in females exceeded that in males (Fig. 1B).

In 2019, Afghanistan, Slovenia, and New Zealand had the highest ASPRs, whereas North Korea and Taiwan had the lowest ASPRs (Fig. 2B, Appendix Table 2). In terms of SDI, the prevalence showed the same trend as incidence; high SDI regions had high ASPRs with a more obvious downward trend (Fig. 3B). The ASPRs for Afghanistan, New Zealand, Slovenia, and Syria were significantly higher than the overall trend, whereas the ASPRs for North Korea and Taiwan were considerably lower (Fig. S2B).

From 1990 to 2019, the most significant reductions in prevalence were seen mainly in developed regions, such as Central and Eastern Europe (Appendix Table 3, Fig. S3B), which also had high ASPRs. Furthermore, the prevalence of facial fractures declined in most countries, as indicated by a negative EAPC for the ASPR. Syria had the highest EAPC, while Liberia had the lowest. The most significant increase in prevalence of 510.4% was seen in Qatar (Fig. S4B, Appendix Table 2).

### Disease burden

Between 1990 and 2019, YLDs increased by 40.2% from 98.1 thousand (95% UI, 58.5–145.8 thousand) to 137.6 thousand (95% UI, 84.3 to 201.4 thousand). However, the age-standardized years lived with disability rate (ASYR) showed a gradual downward trend, from 1.9 (95% UI, 1.2–2.9) per 100,000 to 1.7 (95% UI, 1.1–2.6) per 100,000, with an EAPC of − 0.4 (95% UI, − 0.3 to − 0.5) (Appendix Table 4). Likewise, the male-to-female YLDs ratio for facial fractures remained consistent at around 2:1 over the 30 years (Fig. S1C). Like prevalence, despite consistently higher ASYR in males compared to females in earlier years, the ASYR in females surpassed that in males after the age of 80 years (Fig. 1C).

In 2019, Afghanistan, New Zealand, and Slovenia had the highest ASYRs, while North Korea and Taiwan had the lowest (Fig. 2C, Appendix Table 2). In terms of SDI, ASYR showed a consistent trend across regions, which was similar to that for incidence and prevalence (Fig. 3C).

From 1990 to 2019, the greatest YLD reductions were seen in developed regions, including Central and Eastern Europe (Fig. S3C). The greatest increases in ASYR were seen mainly in developing regions, including the Caribbean, North Africa and the Middle East, South Asia, and Western Sub-Saharan Africa (Appendix Table 4). Qatar had the greatest increase and Eritrea had the greatest decrease in YLDs. Qatar and the Central African Republic had the greatest EAPCs (Fig. S4C, Appendix Table 2).

### Disease burden from different etiologies

**Fig. 4:**
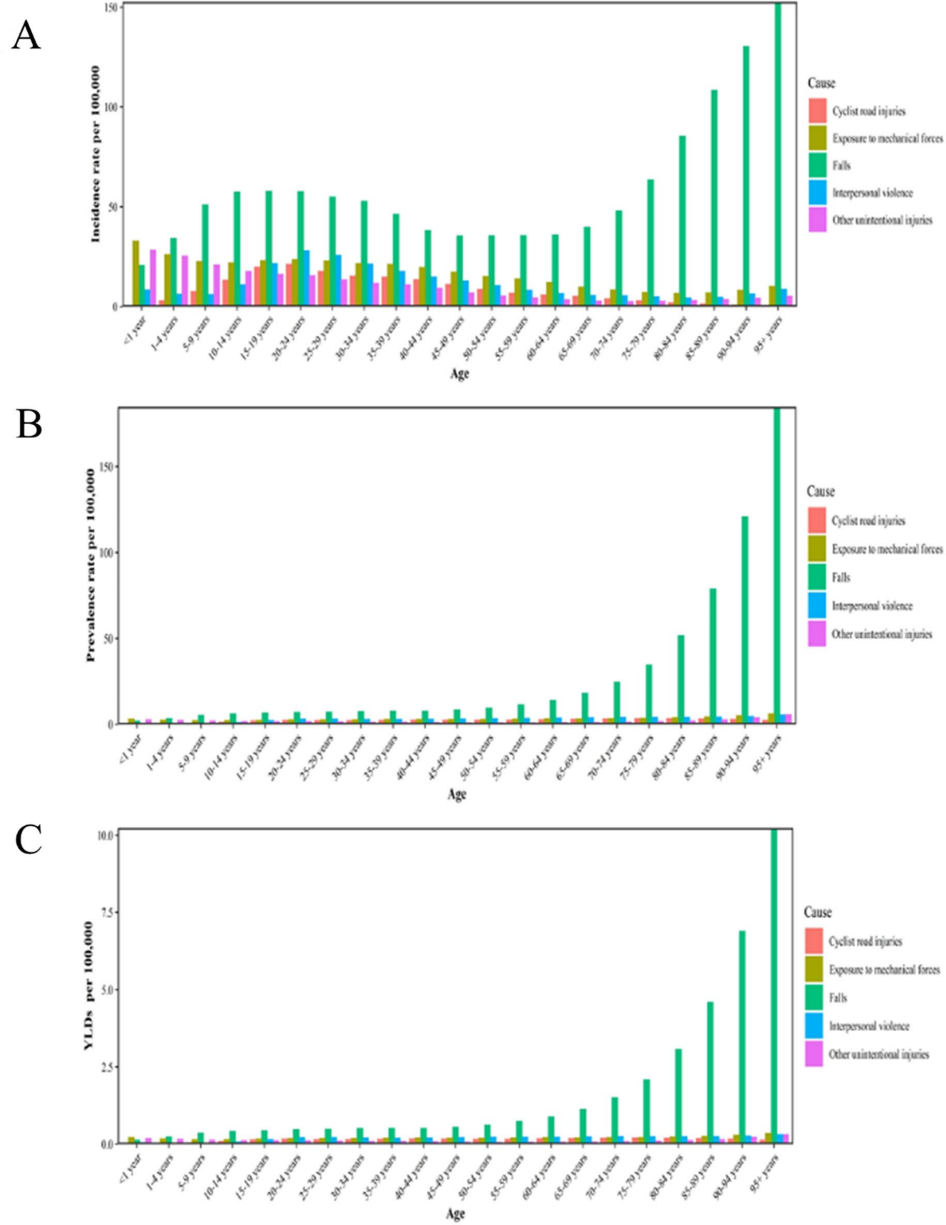
Global disease burden of facial fractures for different age groups by top five causes in 2019. (**A**)Incidence; (**B**) prevalence; (**C**)YLDs

Various causes of facial fractures are reported in GBD 2019; the top eleven causes are shown in Table 1. Falls are the leading cause of facial fractures, with an ASIR of 48.2 thousand (95% UI, 27.3–77.4 thousand) and ASPR of 9.9 thousand (95% UI: 7.7–13.3 thousand). The all-cause ASR showed a downward trend, but the ASR for motorcyclist road injuries showed an increasing trend. At the same time, YLDs and prevalence of falls have an increasing trend with age (Fig. 4B and C). As for the incidence, with the exception of the ˂1 year age group, falls were the leading cause of fractures in all ages. After the age of 40 years, the risk of falls increases with age (Fig. 4A). However, the increase in the incidence of falls was not gradual but had another peak between the ages of 10 and 34 years.

**Table 1:** The twelve leading causes associated with the incidence, prevalence, and YLDs of facial fractures

	Incidence		Prevalence		YLDs	
	ASIR per 100,000 in 2019(95%UI)	EAPC from 1990 to 2019 (95%CI)	ASPR per 100,000 in 2019 (95% UI)	EAPC from 1990 to 2019 (95% CI)	ASYR per 100,000 in 2019 (95% UI)	EAPC from 1990 to 2019 (95% CI)
Falls	48.2 (27.3to77.4)	-0.4 (-0.3to-0.5)	9.9 (7.7to13.3)	-0.3 (-0.2to-0.4)	0.6 (0.4to1.0)	-0.3 (-0.2to-0.4)
Exposure to mechanical forces	20.3 (9.5to37.5)	-0.9 (-0.7to-1)	3.0 (1.9to4.8)	-0.9 (-0.8to-1.1)	0.2 (0.1to0.4)	-0.9 (-0.8to-1.1)
Interpersonal violence	14.4 (8.3to22.4)	-0.6 (-0.6to-0.6)	2.7 (2.0to3.7)	-0.6 (-0.6to-0.7)	0.2 (0.1to0.3)	-0.6 (-0.6to-0.7)
Other unintentional injuries	13.3 (7.5to22.4)	-1.0 (-0.8to-1.1)	1.8 (1.1to2.8)	-1 (-0.9to-1.1)	0.1 (0.1to0.2)	-1.0 (-0.9to-1.1)
Cyclist road injuries	11.8 (6.5to19.4)	0.6 (0.8to0.3)	2.2 (1.6to3.1)	0.4 (0.5to0.2)	0.1 (0.1to0.2)	0.4 (0.6to0.2)
Motor vehicle road injuries	6.2 (3.3to10.6)	-0.2 (-0.2to-0.3)	1.5 (1.2to2)	-0.5 (-0.5to-0.6)	0.1 (0.1to0.2)	-0.5 (-0.4to-0.6)
Animal contact	4.3 (1.9to8.7)	-1.0 (-0.9to-1.2)	0.4 (0.2to0.9)	- (-0.9to-1.2)	0.0 (0.0to0.1)	-1.0 (-0.9to-1.2)
Motorcyclist road injuries	4.1 (2.3to7.2)	1.5 (1.8to1.2)	1.6 (1.3to2)	1.3 (1.6to1)	0.1 (0.1to0.2)	1.3 (1.6to1.0)
Pedestrian road injuries	3.6 (1.8to6.6)	-0.1 (0.0to-0.3)	1.0 (0.8to1.4)	-0.5 (-0.4to-0.6)	0.1 (0.0to0.1)	-0.5 (-0.4to-0.5)
Conflict and terrorism	3.2 (0.9to8.4)	-0.6 (0.8to-2.1)	1.0 (0.3to2.3)	-0.1 (0.6to-0.8)	0.1 (0.0to0.1)	-0.1 (0.7to-0.9)
Foreign body in eyes	3.2 (0.9to8.8)	-1.4 (-1.0to-1.9)	0.4 (0.1to0.9)	-1.3 (-0.9to-1.7)	0.0 (0.0to0.1)	-1.3 (-0.9to-1.7)
Executions and police conflict	0.2 (0.1to0.3)	7.6 (10.1to5.2)	0.0 (0.0to0.1)	6.7 (8.5to4.9)	0.0 (0.0to0.0)	6.7 (8.6to4.9)

*Note* UI, uncertainty interval; CI, confidence interval; YLDs, years lived with disability; EAPC, estimated annual percentage change; ASIR, age-standardized incidence rate; ASPR, age-standardized prevalence rate; ASYR, age-standardized YLD rate

### Relationship between SDI, ASRs, and EAPC

The correlations among SDI, ASRs, and EAPC were analyzed using the Pearson’s correlation coefficient [13]. When 0.9 < *r* < 1, it is highly correlated; When 0.7 < *r* < 0.9, it is strongly correlated; 0.4 < *r* < 0.7 is moderately correlated; 0.2 < *r* < 0.4, indicating weak correlation; 0 < *r* < 0.2, indicating extremely weak or no correlation. The closer the r value is to 1, the stronger the correlation between variables, which means that when the value of one variable increases, the value of another variable often decreases. In 1990, EAPC had a highly negative correlation with ASIR (*p* < 0.01, ρ = −0.44), ASPR (*p* < 0.01, ρ = −0.50), and ASYR (*p* < 0.01, ρ = −0.49), and in 2019, SDI had a highly positive correlation with ASIR (*p* < 0.01, ρ = 0.64), ASPR (*p* < 0.01, ρ = 0.52), and YLDs (*p* < 0.01, ρ = 0.53) (Fig. S2A-C, S5A-C, Fig.S6A-C). But in 2019, there was no correlation with SDI and EAPC of ASIR (*p* = 0.20, ρ = 0.09), of ASPR (*p* = 0.32, ρ = −0.15), or of ASYR (*p* = 0.47, ρ = −0.14). At the same time, in order to verify the effectiveness of the relationship between the validation variables, we calculated the residuals and variances of the variables.

## Discussion

The present study demonstrated changes in the global burden of facial fractures over the past 30 years and their influencing factors. Although the incidence, prevalence, and YLDs of facial fractures increased during the past 30 years, ASRs decreased globally, easpecially in high SDI regions. The present study also analyzed the three variables of facial fractures by age and sex. It is obvious that all three variables were twice as high in males than in females between 1990 and 2019. Facial fracture prevalence and YLDs had a positive correlation with age. Falls were the leading cause of facial fractures and the ASRs of motorcyclist road injuries showed an increasing trend.

Since 1990, the incidence, prevalence, and YLDs of facial fractures showed a global increasing trend. One important reason may be the advancement of facial fracture detection methods. Compared to the previous use of X-ray as the main diagnostic method for fractures, CT and 3D reconstructions, which provide more detailed and rich information, have gradually become the main diagnostic method for facial fractures, especially since the early 1990s. This is likely to have increased pick up rates for facial fractures [11].However, the ASRs of these variables showed a downward trend due to the increasing and aging population. This was consistent with the GBD 2019 Fracture Collaborators [2]. During the study period, the incidence, prevalence, and YLDs of facial fractures remained twice as high in males than in females (Fig. 3A and B, and Fig. 3C). However, not only do males have a higher burden of facial fractures, but also fractures in other parts of the body [2]. This may be because males are more likely to take on high-risk jobs compared to females [15, 16]. In addition, in some regions, a large proportion of males serve in the military, often appearing on the battlefield or participating in military exercises, which may increase the incidence of facial fractures in males. Furthermore, men are more inclined than women to engage in risky behaviors, which can increase the incidence of injuries and accidents, including facial fractures. Preventive measures during engaging in high-risk activities, such as using helmets and protective gear may help reduce the incidence of facial fractures.

Although males had a higher incidence of facial fractures than females, this was not consistent across all age groups. Females older than 75 years had a higher incidence of facial fractures than males of the same age group (Fig. S2A). Previous studies have also demonstrated that older females were more prone to facial fractures than males, possibly because of postmenopausal osteoporosis [17, 18]. Compared to males of the same age, older females have lower bone densities and a greater risk for osteoporosis and osteoarthritis [19].

From 1990 to 2019, ASIR, ASPR, and ASYR showed the same trend relative to the SDI, which was in agreement with a study by Wu et al. [9]. However, in contrast to this study [9], most regions in the present study showed a downward trend. In addition, the correlation between SDI and ASR in different countries in 2019 was analyzed. The downward trend was more pronounced in high SDI areas than in low SDI areas (Fig. 3). The greatest decreases were recorded in the high SDI regions of Central and Eastern Europe (Appendix Table 1). This may be regions with higher SDI pay more attention to public health and have more complete systems and measures to prevent falls, such as seat belts and safety helmets. In addition, these regions have more robust healthcare systems that are better able to detect and treat osteoporosis and fractures. Additionally, according to Manthey et al. [19], alcohol consumption in European countries decreased by 20% between 1990 and 2017. Reduced alcohol consumption and increased awareness of its harms also contributed to reduced ASIR for facial fractures in these regions [20].

However, although high SDI regions showed a decreasing trend of ASRs, these regions had high ASRs. Firstly, this may be because people in high SDI regions were more willing to go to the hospital after a fracture than people in low SDI regions. In low SDI areas, there may be high treatment costs or lack of treatment facilities, leading to under-reporting. Secondly, high SDI countries have more factories and equipment; therefore, traffic accidents and external mechanical forces are major causes of facial fractures in these countries. In addition, Central and Eastern Europe countries have the highest per capita alcohol consumption, reaching a staggering 11.5 L per capita (95% CI, 10.6–12.5) [21]. A strong association between mortality and alcohol consumption has been reported in Eastern and Central Europe [22, 23]. This may be one of the critical reasons for high ASRs in these regions. Developing regions, such as Oceania, North Africa and the Middle East, and the Caribbean, which are middle to high SDI regions showed upward trends in ASR. This may be due to increased alcohol consumption and mechanization as well as a greater number of hospital visits brought about by improved medical care. Although their economic level has improved, there has been no corresponding improvement in medical level, which has caused their incidence to increase.

In addition, SDI was positively associated with the ASRs of incidence, prevalence, and YLDs in the 204 countries and territories. However, four countries, i.e., Afghanistan, New Zealand, Slovenia, and Syria, had ASRs much higher than the overall trends. In contrast, Taiwan and North Korea had ASRs that were much lower than the overall trends. This was consistent with the highest and lowest bone fracture ASRs globally [2]. In general, this may be because Afghanistan, Slovenia, and Syria have high proportions of rural population; the proportion of Afghanistan’s rural population in 2019 was 74% [4], while those in Slovenia and Syria were close to 50%. Previous research has shown that falls are more common among older people in rural areas than non-rural areas [24, 25]. Other studies demonstrated falls were the leading cause of facial fractures in the rural elderly [26]. War has significant effects on a country’s medical care, and can limit the population’s access to medical care. This undoubtedly leads to a significant increase in the YLDs of patients. According to World Bank statistics, the per capita health expenditure in Syria was only $159.58 in 2019, which was far below the average. The combination of war and lack of medical care has made it difficult for Syria to reduce the burden of facial fractures. Part of the reason for New Zealand’s high ASR may be its overweight population, as it has the third highest obesity rate in the world [27]. Previous studies have shown that a high Body Mass Index (BMI) is significantly associated with osteomyelitis, and obesity not only increases the risk of falls but also the risk of fractures [28].

In contrast, the Taiwan region is a high SDI region with very low ASRs. Previous studies have shown that the leading causes of facial fractures in Taiwan were motor vehicle accidents and drunk driving [29, 30]. However, in recent years, Taiwan has changed its traffic laws and implemented policies involving vehicle diversion, helmet use, and increased penalties for drunk driving [27]. The reduced incidence of traffic accidents and facial fractures in Taiwan is possibly a result of these policies. Other contributing factors may be Taiwan’s low crime rate and high healthcare index, the latter of which also significantly reduced the ASYR. In contrast, North Korea’s low ASRs may primarily be due to the damage to North Korea’s public health and medical system caused by US-led economic sanctions [31]. Patients do not have access to medical care and researchers cannot effectively and comprehensively collect information, which may have led to a discrepancy between the statistics and the actual situation. According to a recent study, there are fewer than 200 North Korean health-related publications in five common databases, a situation unique to this country [32].

The most significant increase in incidence of Qatar might be attributed to population growth, which increased from 476,000 in 1990 to 2,832,000 in 2019. The EAPCs of all three variables of Syria were consistently ranked first in the world in our study as a result of the Syrian conflict. The facial fracture incidence rate has increased substantially since 2011 and is still rising [33].

Our study also found that ASRs in 1990 exhibited a significant negative correlation with EAPCs; however, interestingly, EAPCs in 2019 did not correlate with SDI. SDI reflects the average capital income, education level, fertility rate, and basic living standards. While previous studies have shown that socioeconomic status is a risk factor for facial fractures [34], a correlation could not be established because traumatic mechanisms and other risk factors are not associated with SDI. Unlike some region-specific communicable diseases, injuries and facial fractures occur across all geographical locations and income levels. The incidence is more strongly associated with the local condition [35]. As an example, Finland has long winters and falls on ice may explain the high ASRs. This emphasizes that all countries, regardless of the SDI level, should focus on preventing injuries and reducing the frequency of their occurrence.

Our study shows that the leading cause of facial fractures is falls (Table 1), which supports the findings of a previous study [7]. YLDs and prevalence had an increasing trend with age and various age-related comorbidities, such as gait and balance disorders, cognitive impairment, musculoskeletal conditions, and visual impairment, increase the risk of falling [36]. The elderly are at a high risk for osteoporosis, experience more severe injuries compared to younger people when subjected to the same damage factor, and take longer to recover. However, the incidence of falls did not increase with age but showed a higher incidence in people aged 10–34 years. People in this age group are more likely to participate in risky jobs. Alcohol consumption also makes falls more likely in this age group. Previous studies have found that the etiology of facial fractures is affected by local conditions [35], suggesting that the governments should conduct epidemiological surveys and formulate relevant laws, such as using seat belts, to effectively reduce the incidence of facial fractures [37].

In fact, practical policies can effectively reduce the occurrence of fractures and reduce the Disease burden of fractures. A retrospective study in Australia showed that the hospital implementing fracture liaison service (FLS)reduced the incidence rate of serious fractures by 20% compared with the hospital without FLS [38]. Similarly, in a large-scale retrospective survey, it was found that when FLS was introduced, the mortality rate due to osteoporosis decreased [39]. Among them, some countries with high fracture Disease burden also urgently issued corresponding management policies to help establish a sound inquiry and prevention system [40].

Our research results show that to reduce the incidence of facial fractures and Disease burden, policies and implementation should start from the following aspects. Firstly, most fractures occur in the elderly, so it is necessary to raise awareness among the elderly population about screening for osteoporosis and expand the scope of treatment for osteoporosis; Suggesting improving bone strength through exercise and diet throughout the entire life cycle. By consuming nutrients rich in calcium, vitamin D, and other important for bones, the strength of all bones in the body, including facial bones, can be enhanced [41]. In addition, actively engaging in weight-bearing resistance training is beneficial for improving bone density and strength, as well as for facial bones [42]. Secondly, security measures should be strengthened for high-risk workers, and policies should be formulated and implemented to provide them with a safe working environment. Thirdly, for the countries with the highest ASRs, the focus should be on confirming disease policies for preventing fractures. Fourthly, reforming the punishment for traffic violations and reducing alcohol consumption can also help reduce the likelihood of facial fractures. Of course, in addition to designated policies, strong enforcement capabilities are also necessary. Although India has implemented many harsh punishment measures, such as fines and even life imprisonment for drunk driving and unauthorized alcohol sales, its weak implementation has led to high rates of fractures and deaths [43].

However, this study also had certain limitations. First, the data acquisition source was heavily dependent on national data collection. Smaller countries and countries with lower levels of development cannot guarantee the quality of their data. For example, South Africa has one of the highest crime rates in the world. The country has the sixth highest murder rate in the world [44], but the incidence of facial fractures is very low. This is may be missed diagnosis caused by the poor healthcare system in many African countries. Second, differences in disease definitions may lead to statistical differences. Moreover, 95%UI in the data shown in the results section has a significant overlap interval, which may mean that there is no significant difference between the estimated values. Therefore, the findings in our research should be interpreted with caution andreaders should be more cautious when understanding these data.

## Conclusions

In conclusion, facial fractures still impose a major worldwide burden, indicating that the current relevant policies are inadequate. Governments can refer to this study to understand the causes of high and low ASRs in different countries and use this information to formulate more targeted policies.

### Electronic supplementary material

Below is the link to the electronic supplementary material.

Supplementary Material 1

Supplementary Material 2

Supplementary Material 3

Supplementary Material 4

Supplementary Material 5

Supplementary Material 6

Supplementary Material 7

Supplementary Material 8

Supplementary Material 9

Supplementary Material 10

Supplementary Material 11

## Data Availability

The datasets generated for this study can be found in the GBD (http://ghdx.healthdata.org/gbd-results-tool )

## Abbreviations

YLDs: Years lived with disability
ASR: Age-standardized rate
ASIR: Age-standardized incidence rate
ASPR: Age-standardized prevalence rate
ASYR: Age-standardized YLDs rate
GBD: Global Burden of Disease
EAPC: Estimated annual percentage change
IHME: Institute for Health Metrics and Evaluation
GATHER: Guidelines for Reporting Accurate and Transparent Health Estimates
ICD: International Classification of Disease and Injuries
UI: Uncertainty intervals
CI: Certainty intervals
SDI: Socio-demographic index
FLS: Fracture liaison service
