# Supplementary material for: Global, regional, and national burdens of facial fractures: a systematic analysis of the global burden of Disease 2019
Source: BMC Oral Health. 2024 Feb 28;24:282. doi: 10.1186/s12903-024-04048-5 (PMC10900718; doi:10.1186/s12903-024-04048-5)
Supplement: Supplementary file 1 — Supplementary Material 1 [file 12903_2024_4048_MOESM1_ESM.docx]

***Global, regional, and national burdens of facial fractures: a systematic analysis of the Global Burden of Disease 2019***

Appendix table 1 The incidence of facial fractures, and its temporal trends from 1990 to 2019.

|  | 1990 | | 2019 | | 1990 to 2019 | |
| --- | --- | --- | --- | --- | --- | --- |
|  | Incidence Cases  (× 1000)  (95% UI) | ASIR  per 100,000  (95%UI) | Incidence Cases  (× 1000)  (95% UI) | ASIR  per 100,000  (95%UI) | PCC(%) | EAPC  (95%CI)) |
| Global | 8943.7 (7120.5to11371.2) | 161.5 (128.7to204.7) | 10676.3 (8504.3to13455.6) | 138.8 (110.6to174.8) | 19.4 | -0.5 (-0.4to-0.6) |
| **Sex** |  |  |  |  |  |  |
| Female | 2864.0 (2142.8to3825.0) | 104.7 (79.0to139.6) | 3533.6 (2659.6to4706.7) | 93.0 (70.4to123.9) | 23.4 | -0.4 (-0.2to-0.5) |
| Male | 6079.7 (4932.7to7508.4) | 216.6 (175.6to267.4) | 7142.8 (5809.4to8868.8) | 183.4 (149.4to227.7) | 17.5 | -0.5 (-0.4to-0.6) |
| **Region** |  |  |  |  |  |  |
| Andean Latin America | 65.1 (49.6to87.9) | 153.6 (117.6to207.8) | 80.6 (63.7to102.9) | 124.0 (98.0to158.4) | 23.7 | -0.5 (-0.3to-0.7) |
| Australasia | 81.6 (61.5to109.4) | 419.5 (316.4to564.8) | 102.4 (77.2to137.3) | 410.1 (304.4to552.8) | 25.6 | -0.1 (-0.1to-0.2) |
| Caribbean | 51.4(41.4to63.5) | 136.4 (109.7to168.5) | 67.7  (53.8to84.5) | 146.5 (116.0to183.1) | 31.7 | 0.5 (1.4to-0.4) |
| Central Asia | 158.8 (125.4to201.9) | 212.6 (169.3to270.3) | 181.2 (141.2to232.5) | 188.8 (147.4to242.8) | 14.1 | -0.8(-0.5to-1.1) |
| Central Europe | 462.3 (358.6to601.3) | 391.1 (302.9to507.9) | 327.3 (247.5to435.4) | 343.5 (259.9to456.3) | -29.2 | -0.7 (-0.5to-0.8) |
| Central Latin America | 352.7 (273.6to454.8) | 193.6 (150.4to248.3) | 402.1 (312.7to513.5) | 160.5 (124.5to204.6) | 14.0 | 0.1 (0.3to-0.1) |
| Central Sub-Saharan Africa | 61.6 (45.7to88.7) | 98.7 (73.1to138.8) | 116.4 (91.8to146.8) | 80.3 (63.7to100.3) | 88.8 | -1.9 (-0.4to-3.3) |
| East Asia | 1063.8 (773.9to1409.7) | 84.2 (61.4to111.5) | 1368.1 (1013.2to1831.1) | 91.3 (67.3to122.6) | 28.6 | -0.3 (0.1to-0.7) |
| Eastern Europe | 854.0 (671.4to1096.2) | 392.0 (309.3to504.8) | 581.5 (453.7to751.3) | 309.1 (242.0to398.3) | -31.9 | -0.9 (-0.7to-1.0) |
| Eastern Sub-Saharan Africa | 559.4 (292.5to1176.3) | 264.4 (142.8to530.7) | 412.9 (322.3to519.8) | 92.9 (73.2to116.3) | -26.2 | -2.2 (-1.3to-3.1) |
| High-income Asia Pacific | 357.0 (271.6to474.4) | 212.9 (162.6to279.9) | 295.3 (222.0to395.4) | 194.1 (145.0to256.0) | -17.3 | -0.5 (-0.4to-0.6) |
| High-income North America | 589.9 (438.4to779.6) | 214.2 (158.6to284.2) | 630.1 (468.4to828.9) | 178.7 (132.3to233.8) | 6.8 | -1.1 (-0.8to-1.5) |
| North Africa and Middle East | 602.1 (472.7to764.4) | 158.4 (125.2to200.3) | 1005.2 (767.6to1336.2) | 159.6 (121.7to213.2) | 67 | 1.0 (1.5to0.6) |
| Oceania | 5.8 (4.7to7.2) | 85.4 (69.1to105.5) | 13.3 (10.4to16.7) | 96.1 (75.3to120.1) | 127.9 | 0.1 (0.6to-0.3) |
| South Asia | 1719.3 (1372.1to2161.0) | 153.1 (123.6to191.0) | 2776.8 (2197.3to3531.3) | 150.5 (119.3to191.0) | 61.5 | -0.1 (0.1to-0.2) |
| Southeast Asia | 584.3 (464.4to734.6) | 116.8 (93.4to146.4) | 680.7 (541.4to854.2) | 100.6 (79.7to126.7) | 16.5 | -0.5 (-0.3to-0.8) |
| Southern Latin America | 118.7  (91.9to154.4) | 235.2 (182.3to306.4) | 149.1 (115.3to195.8) | 234.9 (181.2to308.0) | 25.7 | -0.1 (-0.1to-0.2) |
| Southern Sub-Saharan Africa | 59.2  (45.8to75.9) | 108.3 (83.3to137.5) | 71.7 (55.8to90.1) | 88.2 (69.0to110.0) | 21.1 | -0.6 (-0.5to-0.7) |
| Tropical Latin America | 266.7 (196.4to356.3) | 163.2 (120.6to217.5) | 312.1 (230.5to414.1) | 140.9 (104.2to188.7) | 17 | -0.5 (-0.4to-0.5) |
| Western Europe | 756.9 (579.8to987.9) | 216.1 (164.7to283.4) | 718.8 (536.5to972.7) | 197.9 (145.3to267.9) | -5 | -0.5 (-0.4to-0.6) |
| Western Sub-Saharan Africa | 172.9 (130.9to231.8) | 84.9 (64.3to111.7) | 383.2 (293.8to492.6) | 80.8 (62.2to102.8) | 121.6 | -0.1 (0.2to-0.4) |
| Note: ASIR, age-standardized prevalence rate; PCC, percent change in cases; EAPC, estimated annual percentage change. | | | | | | |
