# Supplementary material for: Global, regional, and national burdens of facial fractures: a systematic analysis of the global burden of Disease 2019
Source: BMC Oral Health. 2024 Feb 28;24:282. doi: 10.1186/s12903-024-04048-5 (PMC10900718; doi:10.1186/s12903-024-04048-5)
Supplement: Supplementary file 2 — Supplementary Material 2 [file 12903_2024_4048_MOESM2_ESM.docx]

Appendix table 2 Three countries with the largest and lowest number of incidence, prevalence, or YLDs.

| Measure |  |  | Top three countries |  |  |  |  |  | Bottom three countries |  |  |  |  |
| --- | --- | --- | --- | --- | --- | --- | --- | --- | --- | --- | --- | --- | --- |
| 2019ASR (per 100,000 people) | | |  |  |  |  |  |  |  |  |  |  |  |
| ASIR | New Zealand | 464.2 | Slovenia | 401.1 | Australia | 400.2 | Liberia | 65.5 | Taiwan (Province of China) | 62.9 | Democratic People's Republic of Korea | 59.2 |  |
| ASPR | Afghanistan | 83.7 | New Zealand | 71.9 | Slovenia | 67.4 | Kiribati | 12.4 | Taiwan (Province of China) | 12.3 | Democratic People's Republic of Korea | 11.4 |  |
| ASYR | Afghanistan | 5.2 | New Zealand | 4.7 | Slovenia | 4.4 | Kiribati | 0.8 | Taiwan (Province of China) | 0.8 | Democratic People's Republic of Korea | 0.7 |  |
| 1990 to 2019 percent change in cases | | |  |  |  |  |  |  |  |  |  |  |  |
| Incidence | Qatar | 497.3 | Yemen | 496.2 | Afghanistan | 411.9 | Ethiopia | -69.5 | Liberia | -79.4 | Eritrea | -82.1 |  |
| Prevalence | Qatar | 510.4 | Yemen | 432.3 | United Arab Emirates | 412.7 | Ethiopia | -54.7 | Liberia | -60.9 | Eritrea | -63.7 |  |
| YLDs | Qatar | 510.4 | Yemen | 433.6 | United Arab Emirates | 409.0 | Ethiopia | -55.6 | Liberia | -62.3 | Eritrea | -65.1 |  |
| EAPC | | |  |  |  |  |  |  |  |  |  |  |  |
| Incidence | Syrian | 8.2 | Central African Republic | 3.8 | Yemen | 3.1 | Timor-Leste | -5.1 | Burundi | -5.8 | Liberia | -6.3 |  |
| Prevalence | Syrian | 7.5 | Central African Republic | 3.3 | Yemen | 2.2 | Timor-Leste | -2.4 | Eritrea | -3.1 | Liberia | -3.6 |  |
| YLDs | Syrian | 7.5 | Central African Republic | 3.3 | Yemen | 2.2 | Angola | -2.5 | Eritrea | -3.2 | Liberia | -3.7 |  |
| Note: ASIR, age-standardized prevalence rate; ASPR, age-standardized prevalence rate; YLDs, years lived with disability; ASYR, age-standardized YLDs rate; EAPC, estimated annual percentage change. | | | | | | | | | | | | | |
