# Supplementary material for: Global, regional, and national burdens of facial fractures: a systematic analysis of the global burden of Disease 2019
Source: BMC Oral Health. 2024 Feb 28;24:282. doi: 10.1186/s12903-024-04048-5 (PMC10900718; doi:10.1186/s12903-024-04048-5)
Supplement: Supplementary file 3 — Supplementary Material 3 [file 12903_2024_4048_MOESM3_ESM.docx]

Appendix table 3. The prevalence of facial fractures and its temporal trends from 1990 to 2019.

|  | 1990 | | 2019 | | 1990 to 2019 | |  |
| --- | --- | --- | --- | --- | --- | --- | --- |
|  | Prevalence Cases  (× 1000)  (95% UI) | ASPR  per 100,000  (95%UI) | Prevalence Cases  (× 1000)  (95% UI) | ASPR  Per 100,000  (95%UI) | PCC(%) | EAPC  (95% CI)) |  |
| Global | 1502.8 (1242.6to1823.6) | 30.1 (25.4to35.9) | 2131.6 (1815.7to2509.6) | 27.0 (23.0to31.9) | 41.8 | -0.4 (-0.3to-0.5) |  |
| **Sex** |  |  |  |  |  |  |  |
| Female | 513.4 (424.0to641.1) | 20.7 (17.4to25.4) | 766.8 (648.1to925.2) | 19.1 (16.0to23.2) | 49.4 | -0.3 (-0.2to-0.4) |  |
| Male | 989.4 (821.7to1192.7) | 39.5 (33.4to46.9) | 1364.8 (1156.6to1599.5) | 35.0 (29.7to40.9) | 37.9 | -0.4 (-0.4to-0.5) |  |
| **Region** |  |  |  |  |  |  |  |
| Andean Latin America | 9.1 (7.1to12.0) | 24.7 (19.9to31.4) | 13.2 (10.8to16.3) | 21.0 (17.3to25.7) | 44.7 | -0.4 (-0.3to-0.6) |  |
| Australasia | 13.1 (10.6to16.7) | 64.5 (51.3to83.2) | 19.2 (16.1to23.8) | 63.6 (51.0to82.0) | 46.6 | -0.1 (-0.0to-0.1) |  |
| Caribbean | 8.2 (6.7to9.9) | 23.9 (20.1to28.7) | 13.2 (11.0to15.8) | 27.3 (22.5to32.9) | 61.1 | 0.8 (1.4to0.1) |  |
| Central Asia | 23.8 (19.1to29.5) | 35.4 (29.1to43.0) | 29.4 (23.9to36.3) | 31.9 (26.2to39.0) | 23.9 | -0.6 (-0.4to-0.8) |  |
| Central Europe | 83.1 (69.2to101.3) | 66.1 (54.4to81.6) | 70.9 (60.6to84.4) | 57.5 (46.9to71.6) | -14.7 | -0.6 (-0.5to-0.7) |  |
| Central Latin America | 53.2 (42.9to66.3) | 34.7 (29.0to42.0) | 70.6 (58.5to85.6) | 28.4 (23.5to34.5) | 32.8 | 0.0 (0.3to-0.2) |  |
| Central Sub-Saharan Africa | 9.4 (7.0to13.0) | 19.2 (15.2to25.6) | 20.5 (15.7to27.7) | 18.9 (14.8to26.1) | 117.3 | -0.8 (0.1to-1.7) |  |
| East Asia | 180.9 (146.0to224.4) | 16.0 (13.1to19.5) | 307.7 (261.8to366.0) | 18.1 (15.1to21.7) | 70.1 | -0.1 (0.3to-0.4) |  |
| Eastern Europe | 160.1 (135.3to190.6) | 68.0 (56.7to82.0) | 125.4 (107.7to146.7) | 54.2 (45.0to65.4) | -21.7 | -0.8 (-0.7to-0.9) |  |
| Eastern Sub-Saharan Africa | 71.3 (40.5to132.8) | 39.2 (24.6to68.8) | 70.9 (55.6to94.0) | 21.5 (17.1to30.3) | -0.6 | -1.2 (-0.7to-1.8) |  |
| High-income Asia Pacific | 62.5 (51.4to76.9) | 35.1 (28.5to43.6) | 70.3 (60.7to82.7) | 31.9 (25.9to40.1) | 12.6 | -0.5 (-0.4to-0.6) |  |
| High-income North America | 114.7 (96.3to137.2) | 38.5 (31.7to46.9) | 151.0 (131.4to176.5) | 34.1 (28.5to41.1) | 31.7 | -0.8 (-0.5to-1.1) |  |
| North Africa and Middle East | 97.1 (72.9to134.9) | 31.3 (23.7to46.2) | 177.1 (135.7to247.2) | 30.1 (23.2to42.2) | 82.4 | 0.4 (0.7to0.1) |  |
| Oceania | 0.9 (0.7to1.1) | 16.2 (13.8to19.0) | 2.2 (1.8to2.6) | 18.7 (15.8to22.1) | 142.1 | 0.3 (0.6to0.1) |  |
| South Asia | 279.7 (227.9to334.4) | 30.5 (25.9to35.6) | 536.1 (449.6to634.9) | 32.1 (27.3to37.4) | 91.6 | 0.2 (0.3to0.0) |  |
| Southeast Asia | 91.8 (74.8to113.1) | 21.7 (18.3to26.0) | 129.3 (108.8to153.5) | 19.5 (16.4to23.0) | 40.8 | -0.4 (-0.3to-0.6) |  |
| Southern Latin America | 18.6 (15.0to23.2) | 37.8 (30.7to46.9) | 25.5 (21.0to31.4) | 37.5 (30.4to46.7) | 37.5 | -0.2 (-0.1to-0.2) |  |
| Southern Sub-Saharan Africa | 9.8 (8.1to12.0) | 21.8 (18.4to25.9) | 12.7 (10.6to15.4) | 17.2 (14.6to20.5) | 29.4 | -0.7 (-0.5to-0.8) |  |
| Tropical Latin America | 41.5 (32.9to52.3) | 29.3 (24.0to36.0) | 58.4 (48.1to71.6) | 25.3 (20.6to31.2) | 40.7 | -0.5 (-0.4to-0.6) |  |
| Western Europe | 147.9 (124.8to178.6) | 36.4 (29.7to45.1) | 167.5 (144.3to198.3) | 33.3 (26.8to41.9) | 13.2 | -0.5 (-0.4to-0.6) |  |
| Western Sub-Saharan Africa | 26.3 (20.8to33.3) | 16.5 (13.9to19.9) | 60.6 (48.9to74.7) | 16.7 (14.1to19.7) | 130.9 | 0.1 (0.2to-0.1) |  |
| Note: ASPR, age-standardized prevalence rate; PCC, percent change in  cases; EAPC, estimated annual percentage change. | | | | | | |  |
|  |  |  |  |  |  |  |  |
