# Supplementary material for: Global, regional, and national burdens of facial fractures: a systematic analysis of the global burden of Disease 2019
Source: BMC Oral Health. 2024 Feb 28;24:282. doi: 10.1186/s12903-024-04048-5 (PMC10900718; doi:10.1186/s12903-024-04048-5)
Supplement: Supplementary file 4 — Supplementary Material 4 [file 12903_2024_4048_MOESM4_ESM.docx]

Appendix table 4 The YLDs of facial fractures, and its temporal trends from 1990 to 2019.

|  | 1990 | | 2019 | | 1990 to 2019 | |
| --- | --- | --- | --- | --- | --- | --- |
|  | YLDs  (×1000)  (95% UI) | ASYR  Per 100,000  (95%UI) | YLDs  (× 1000)  (95% UI) | ASYR  per 100,000  (95%UI) | PCC(%) | EAPC  (95% CI)) |
| Global | 98.1(58.5to145.8) | 1.9 (1.2to2.9) | 137.6 (84.3to201.4) | 1.7 (1.1to2.6) | 40.2 | -0.4 (-0.3to-0.5) |
| **Sex** |  |  |  |  |  |  |
| Female | 33.3 (19.8to49.6) | 1.3 (0.8to2.0) | 49.1 (30.2to72.0) | 1.2 (0.8to1.8) | 47.2 | -0.3(-0.2to-0.5) |
| Male | 64.8 (38.6to95.8) | 2.6 (1.6to3.8) | 88.5 (54.2to129.9) | 2.3 (1.4to3.3) | 36.6 | -0.4 (-0.4to-0.5) |
| **Region** |  |  |  |  |  |  |
| Andean Latin America | 0.6 (0.4to0.9) | 1.6 (1.0to2.5) | 0.9 (0.5to1.3) | 1.4 (0.8to2.0) | 43.3 | -0.4 (-0.3to-0.6) |
| Australasia | 0.9 (0.5to1.3) | 4.2 (2.5to6.4) | 1.2 (0.7to1.8) | 4.2 (2.4to6.3) | 44.6 | -0.1 (-0.0to-0.1) |
| Caribbean | 0.5 (0.3to0.8) | 1.6 (0.9to2.3) | 0.9 (0.5to1.2) | 1.8 (1.1to2.6) | 58.4 | 0.7 (1.4to0.1) |
| Central Asia | 1.6 (0.9to2.3) | 2.3 (1.4to3.5) | 1.9 (1.2to2.9) | 2.1 (1.3to3.1) | 23.6 | -0.6 (-0.4to-0.8) |
| Central Europe | 5.4 (3.2to8.0) | 4.3 (2.6to6.4) | 4.5 (2.8to6.7) | 3.7 (2.2to5.6) | -15.6 | -0.6 (-0.5to-0.7) |
| Central Latin America | 3.5 (2.1to5.3) | 2.3 (1.4to3.4) | 4.6 (2.8to6.9) | 1.8 (1.1to2.8) | 31.5 | 0.0 (0.3to-0.2) |
| Central Sub-Saharan Africa | 0.6 (0.4to1.0) | 1.2 (0.8to1.9) | 1.3 (0.8to2.0) | 1.2 (0.7to1.8) | 115.1 | -0.9 (0.1to-1.8) |
| East Asia | 11.9 (7.0to18.4) | 1.0 (0.6to1.6) | 19.9 (12.0to29.9) | 1.2 (0.7to1.8) | 67.0 | -0.1 (0.3to-0.4) |
| Eastern Europe | 10.4 (6.3to15.4) | 4.4 (2.6to6.6) | 8.1 (4.9to11.9) | 3.5 (2.1to5.2) | -22.2 | -0.8 (-0.7to-0.9) |
| Eastern Sub-Saharan Africa | 4.7 (2.2to9.7) | 2.6 (1.3to5.0) | 4.6 (2.9to6.8) | 1.4 (0.9to2.0) | -2.1 | -1.3 (-0.7to-1.8) |
| High-income Asia Pacific | 4.1 (2.4to6.1) | 2.3 (1.4to3.5) | 4.5 (2.7to6.7) | 2.1 (1.2to3.2) | 10.3 | -0.5 (-0.4to-0.6) |
| High-income North America | 7.4 (4.4to11.1) | 2.5 (1.5to3.8) | 9.5 (5.9to14.1) | 2.2 (1.3to3.3) | 28.8 | -0.8 (-0.5to-1.2) |
| North Africa and Middle East | 6.3 (3.8to9.6) | 2.0 (1.2to3.1) | 11.5 (7.0to17.4) | 1.9 (1.2to2.9) | 81.7 | 0.5 (0.8to0.2) |
| Oceania | 0.1 (0.0to0.1) | 1.1 (0.6to1.5) | 0.1 (0.1to0.2) | 1.2 (0.7to1.8) | 141 | 0.3 (0.6to0.0) |
| South Asia | 18.3 (10.7to27.1) | 2.0 (1.2to2.9) | 34.6 (21.2to51.5) | 2.1 (1.3to3.0) | 89.4 | 0.1 (0.3to-0.0) |
| Southeast Asia | 6.0 (3.7to9.0) | 1.4 (0.9to2.1) | 8.4 (5.2to12.3) | 1.3 (0.8to1.8) | 39.4 | -0.4 (-0.3to-0.6) |
| Southern Latin America | 1.2 (0.7to1.8) | 2.5 (1.5to3.7) | 1.7 (1.0to2.5) | 2.5 (1.5to3.8) | 36.6 | -0.2 (-0.1to-0.2) |
| Southern Sub-Saharan Africa | 0.6 (0.4to1.0) | 1.4 (0.9to2.1) | 0.8 (0.5to1.2) | 1.1 (0.7to1.7) | 28.8 | -0.7 (-0.5to-0.8) |
| Tropical Latin America | 2.7 (1.6to4.2) | 1.9 (1.1to2.9) | 3.8 (2.3to5.8) | 1.6 (1.0to2.5) | 39.3 | -0.5 (-0.4to-0.6) |
| Western Europe | 9.6 (5.8to14.2) | 2.4 (1.4to3.6) | 10.7 (6.5to15.9) | 2.2 (1.3to3.3) | 11.6 | -0.5 (-0.4to-0.6) |
| Western Sub-Saharan Africa | 1.7 (1.0to2.6) | 1.1 (0.6to1.6) | 4.0 (2.4to5.9) | 1.1 (0.7to1.6) | 130.7 | 0.1 (0.2to-0.1) |
| Note: ASYR, age-standardized YLDs rate;  YLDs, years lived with disability; PCC, percent change in cases; EAPC,  estimated annual percentage change. | | | | | | |
