# Supplementary figures and images for: Global, regional, and national burdens of facial fractures: a systematic analysis of the global burden of Disease 2019
Source: BMC Oral Health. 2024 Feb 28;24:282. doi: 10.1186/s12903-024-04048-5 (PMC10900718; doi:10.1186/s12903-024-04048-5)

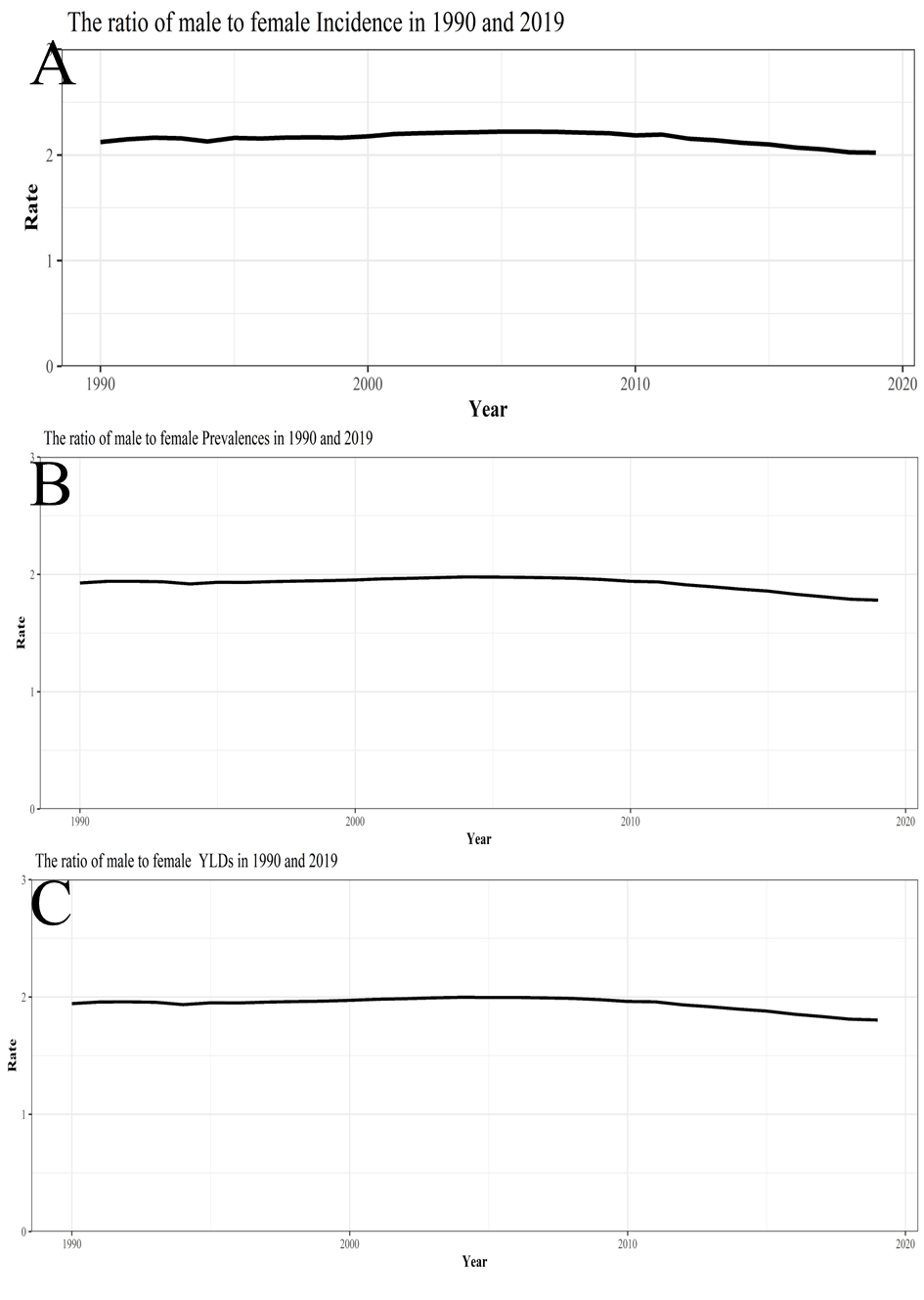

Supplement: Supplementary file 5 — Supplementary Material 5 [file 12903_2024_4048_MOESM5_ESM.tif]

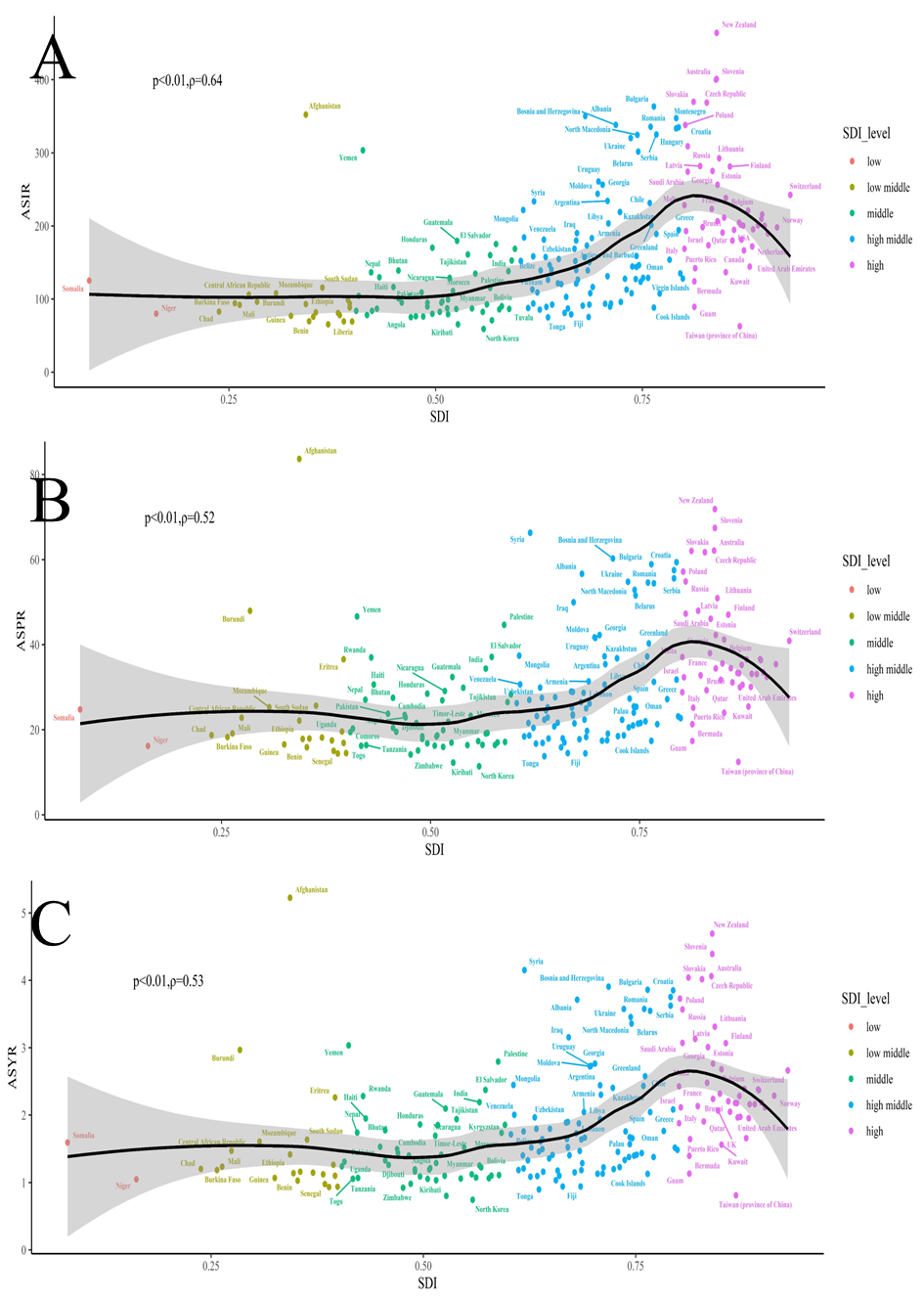

Supplement: Supplementary file 6 — Supplementary Material 6 [file 12903_2024_4048_MOESM6_ESM.tif]

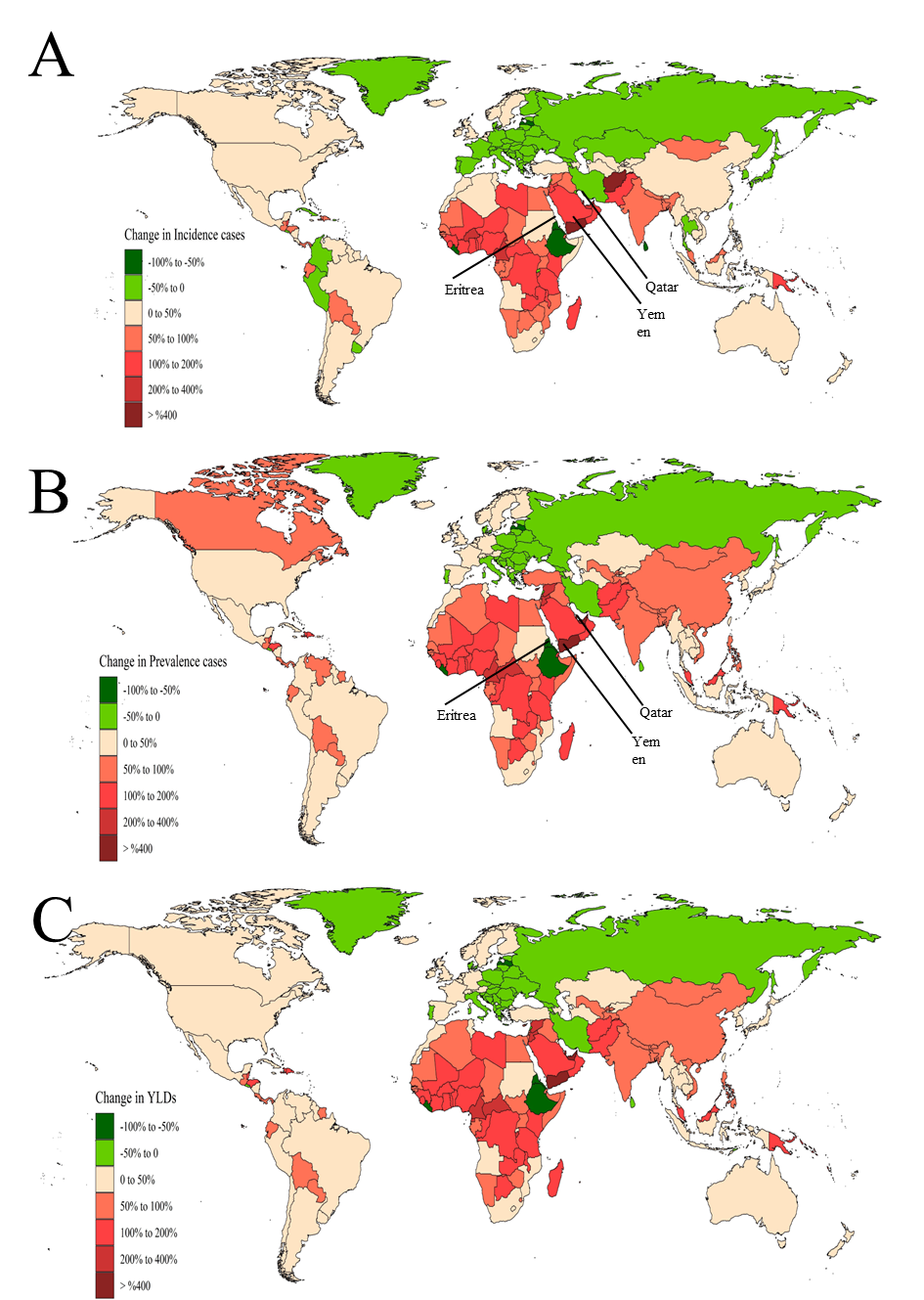

Supplement: Supplementary file 7 — Supplementary Material 7 [file 12903_2024_4048_MOESM7_ESM.tif]

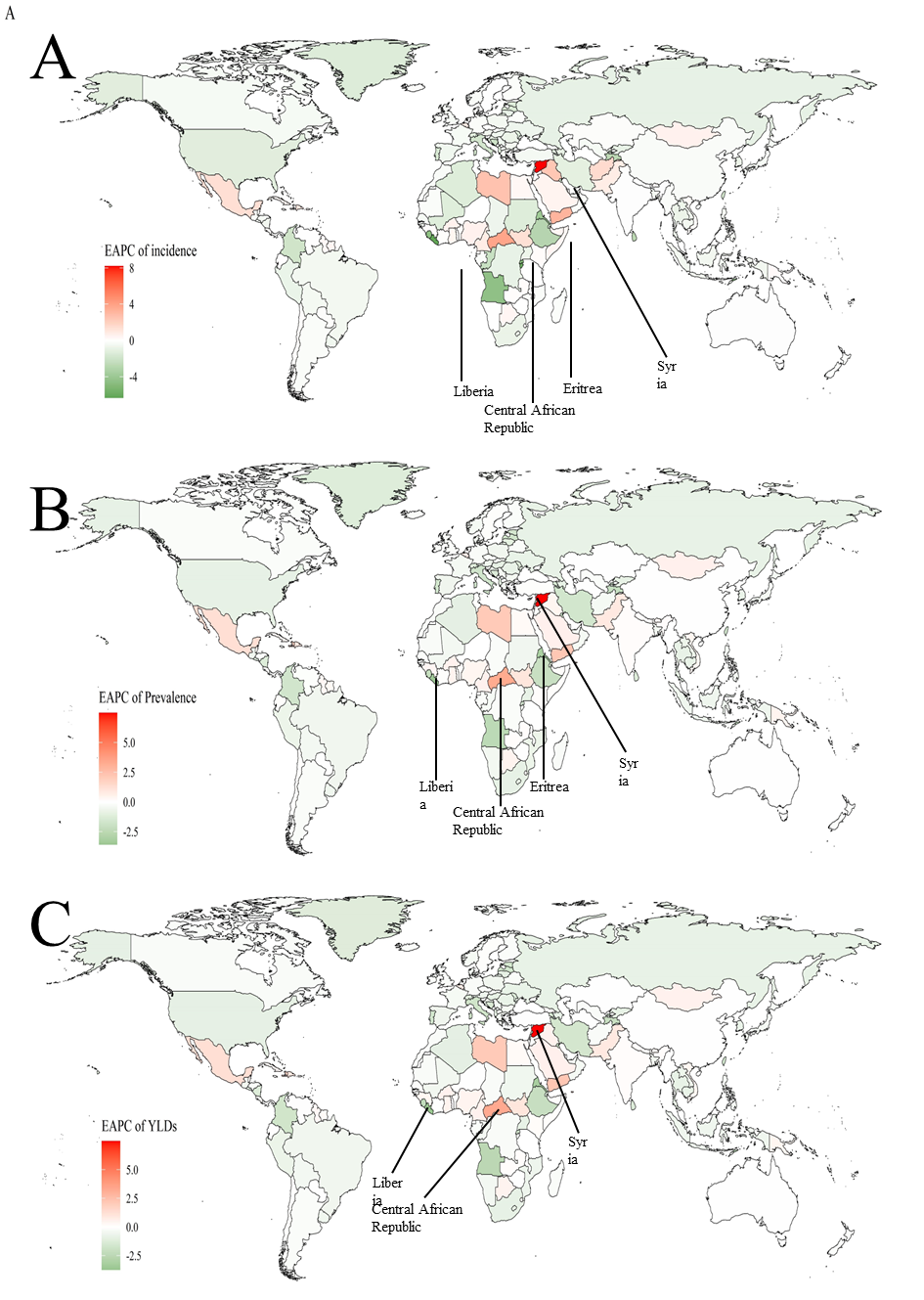

Supplement: Supplementary file 8 — Supplementary Material 8 [file 12903_2024_4048_MOESM8_ESM.tif]

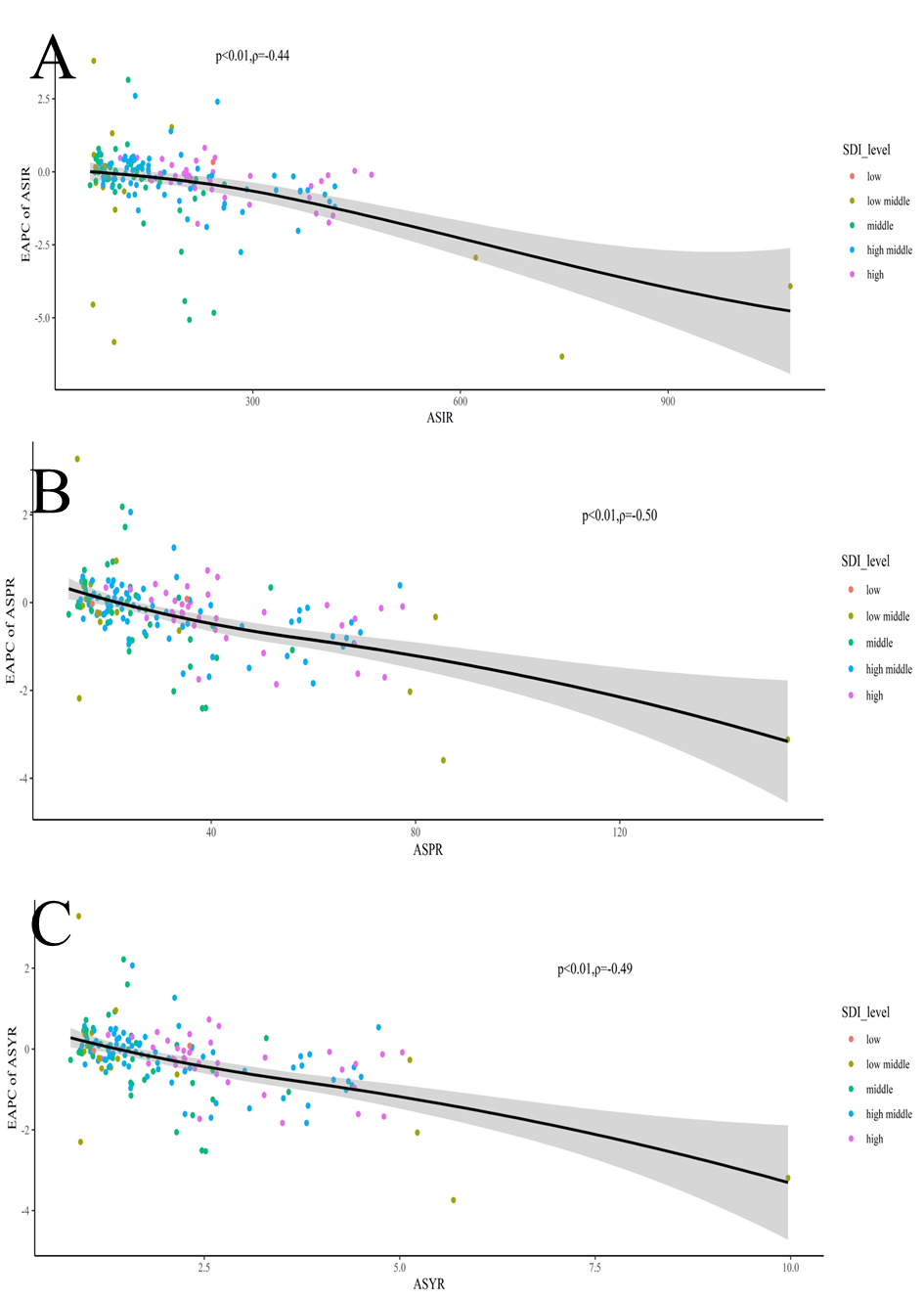

Supplement: Supplementary file 9 — Supplementary Material 9 [file 12903_2024_4048_MOESM9_ESM.tif]

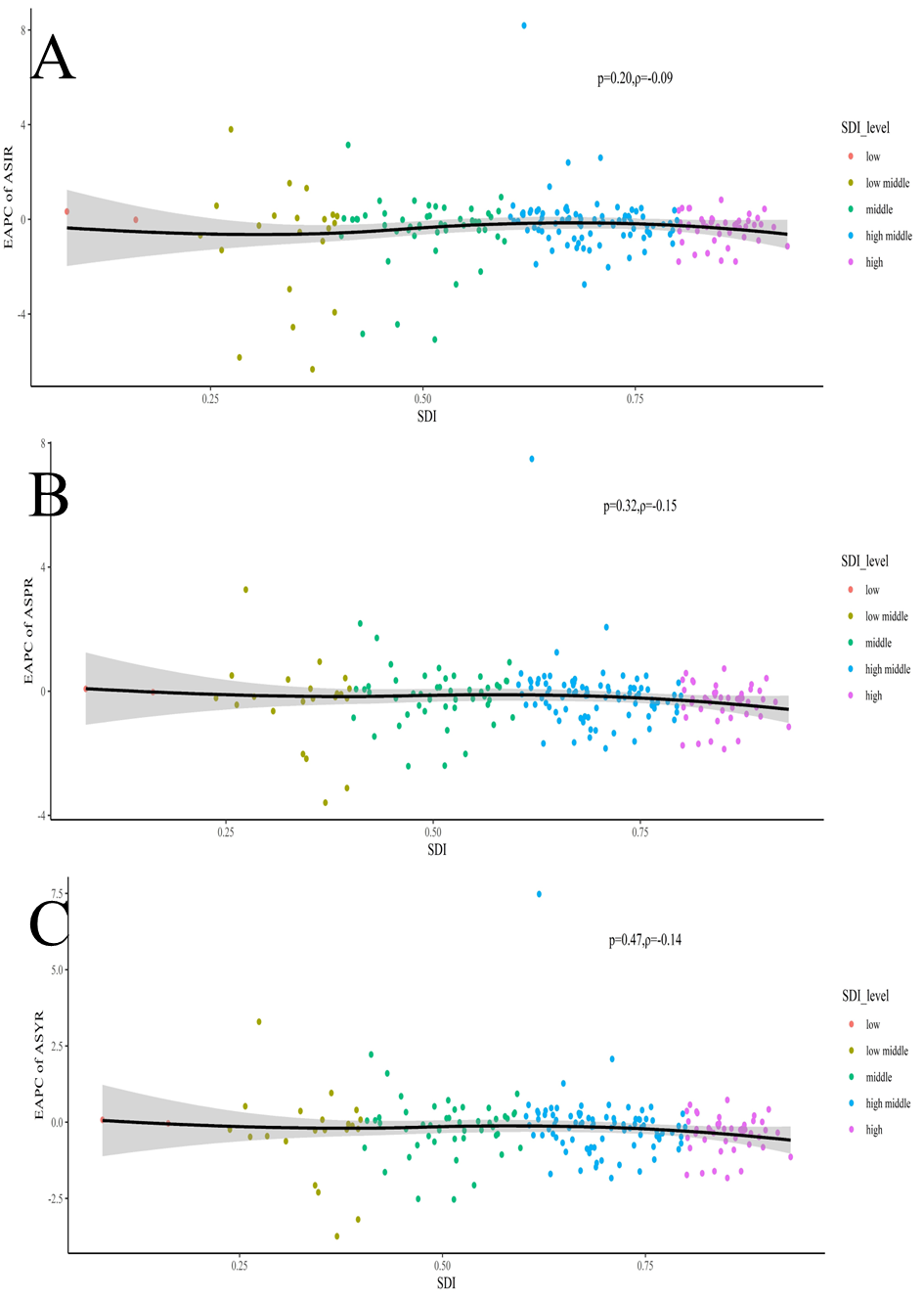

Supplement: Supplementary file 10 — Supplementary Material 10 [file 12903_2024_4048_MOESM10_ESM.tif]
