# Supplementary material for: Global, regional, and national burdens of facial fractures: a systematic analysis of the global burden of Disease 2019
Source: BMC Oral Health. 2024 Feb 28;24:282. doi: 10.1186/s12903-024-04048-5 (PMC10900718; doi:10.1186/s12903-024-04048-5)
Supplement: Supplementary file 11 — Supplementary Material 11 [file 12903_2024_4048_MOESM11_ESM.doc]

**Supplementary figure and appendix table legends**

**Appendix Table 1.** The incidence of facial fractures and its temporal trends from 1990 to 2019.

**Appendix Table 2.** Three countries with the largest and lowest number of incidence,

prevalence, or YLDs.

**Appendix Table 3.** The prevalence of facial fractures and its temporal trends from 1990 to 2019.

**Appendix Table 4.** The YLDs of facial fractures and its temporal trends from 1990 to 2019.

**Figure legends**

**Figure S1.** The ratio of male to female of global disease burden of facial fracture, 1990–2019. (A)Incidence; (B) prevalence; (C)YLDs.

**Figure S2.** The association between the ASRs of facial fractures and the SDI among 204 countries and territories between 1990 and 2019. (A) ASIR; (B) ASPR; (C)ASYR.

**Figure S3.** The relative change percent of facial fractures among regions, 1990–2019. (A)Incidence; (B) prevalence; (C)YLDs.

**Figure S4.** The EAPC of the ASRs of facial fractures from 1990 to 2019. (A) ASIR; (B) ASPR; (C)ASYR.

**Figure S5.** The correlation between the EAPCs and ASRs of facial fractures in 1990 among 204 countries and territories. (A) ASIR; (B) ASPR; (C)ASYR.

**Figure S6.** The correlation between the EAPC of the ASRs of facial fractures and the SDI in 2019 among 204 countries and territories. (A) ASIR; (B) ASPR; (C)ASYR.
